# Decreased expression of endothelial cell specific molecule-1 in lung tissue in emphysematous mice and stable COPD patients

**DOI:** 10.22038/ijbms.2020.44243.10384

**Published:** 2020-12

**Authors:** Yan Zhang, Ping Chen, Shan Cai, Jinhua Li, Yan Chen

**Affiliations:** 1 Division of Pulmonary and Critical Care Medicine, The Second Xiangya Hospital, Central South University, Changsha 410011, Hunan, China; 2 Research Unit of Respiratory Diseases, Central South University, Changsha 410011, Hunan, China; 3 Diagnosis and Treatment Center of Respiratory Diseases, Central South University, Changsha 410011, Hunan, China

**Keywords:** Alveolar, Apoptosis, Chronic obstructive - pulmonary disease, Endothelial cell specific - molecule-1, Hepatocyte growth factor, Vascular endothelial growth- factor

## Abstract

**Objective(s):**

Apoptosis of pulmonary alveolar septal cells is a pathogenesis characteristic of chronic obstructive pulmonary disease (COPD). Endothelial cell specific molecule-1 (ESM-1) plays an important role in apoptosis of cells. Here, we aimed to determine whether ESM-1 can involve in cell apoptosis in emphysematous mice and stable COPD patients. The sample size of patients was small, so two separate models were studied.

**Materials and Methods:**

At day 0, 11, and 22, murine were injected IP with 0.3 ml of PBS/Cigarette smoke extract, and euthanized at day 28. Lung tissues from 20 stable COPD patients and 12 Controls were evaluated. Serum was obtained from 25 stable COPD patients and 12 healthy Controls. Pulmonary function, pathology, pulmonary apoptosis index (AI), expression of vascular endothelial growth factor (VEGF), hepatocyte growth factor (HGF) and ESM-1 in lung tissue, and concentration of ESM-1 in serum were tested.

**Results:**

Protein expression of ESM-1, VEGF and HGF were decreased significantly in emphysematous mice (*P*<0.05), while AI was increased (*P*<0.05). Correlation analysis indicated that association between AI and ESM-1 was negative (*P*<0.01), VEGF and ESM-1 was positive (*P*<0.01), and HGF and ESM-1 was positive (*P*<0.01). In stable COPD patients, we proved that ESM-1, VEGF and HGF were decreased significantly, while AI was increased (*P*<0.05). Correlation between AI and ESM-1 was negative (*P*<0.01), VEGF and ESM-1 was positive (*P*<0.01), and HGF and ESM-1 was positive (*P*<0.01).

**Conclusion:**

ESM-1 expression decreased and AI increased in emphysematous mice and stable COPD patients. Findings suggested that ESM-1 may be involved in anti-apoptotic therapy of COPD.

## Introduction

Chronic obstructive pulmonary disease (COPD) is a chronic airway disease characterized by incomplete reversible airflow limitation. Cigarette smoking is one risk factor for COPD development and progression throughout the world (1). Cigarette-induced abnormal apoptosis is one of pathogenic mechanisms of COPD (1-5). Vascular endothelial growth factor (VEGF) and hepatocyte growth factor (HGF) are negatively associated with apoptosis of alveolar septal cells in COPD (6,7).

Endothelial cell specific molecule-1 (ESM-1) is a proteoglycan, mainly secreted by endothelial cells and epithelial cells in lung tissue (8). Human ESM-1 is synthesized as a 20 kDa protein. The posttranslational modified isoform is approximately 30 kDa in size (8), and consists of a single dermatan sulfate (DS) chain that contains 4-O sulfated N-acetyl galactosamine with α-iduronate (9). Evidences (10-12) suggested that ESM-1 plays an important part in inflammation and upregulates expression of adhesion molecules and cytoskeleton rearrangement in endothelium. ESM-1 interacts with HGF, through its glycan chain, and dose-dependently increases HGF-mediated proliferation of cells (9). ESM-1 expression is positively correlated with VEGF expression, and VEGF represents a powerful inducer of ESM-1 secretion (11). ESM-1 leads to angiogenesis and induces proliferation of cancerous cells via HGF/SF and VEGF pathways, and suppresses apoptosis (12). The involvement of ESM-1 in angiogenesis and proliferation of cells, its preferential expression in lung, and relationship to growth factors let us to explore the relation between expression of ESM-1 and apoptosis in COPD. Our purpose in this study was to determine whether ESM-1 can involve in cell apoptosis in emphysematous mice and stable COPD patients. The sample size of patients was small, so two separate models were studied in the study.

## Materials and Methods

Each experiment was replicated for three times, and the extent of variation from experiment to experiment was less than 5%. This study (two models) was approved by Ethics Committee of Second Xiangya Hospital (application number 2008s061), and participants gave their written informed consents. 

***Emphysematous mice model***

One cigarette was burned and collected in a tube containing 2 ml of phosphate-buffered saline (PBS) (5). Six week male SPF BALB/c mice (21–23 grams) were randomly divided into PBS-group (n=7) and cigarette smoke extract (CSE)-group (n=14). Mice were fed in Second Xiangya Hospital Experimental Animal Center, maintained at 21-22 ^°^C and 50%-70% humidity in a room with light-dark cycle and air purification. 

Our team previously established emphysematous model by injection of CSE (2, 3, 5), and the results showed decline in pulmonary function, alveolus destruction, apoptosis induction, inflammatory infiltration, reduction in biological antioxidant activity, and activation of matrix metalloproteinase in mice. In this study, mice were injected IP with 0.3 ml CSE at day 0, 11, and 22 to establish emphysematous model (5), while other mice were injected IP with 0.3 ml if PBS at the same time (5). All mice were drawn blood from the caudal vein under anaesthesia at day 28, and then were euthanized under anaesthesia (5). 

***Control participants and patients with chronic obstructive pulmonary disease***

Lung tissue was examined from patients undergoing lung resection for lung cancer, 12 Control participants (9 men and 3 women; 63.4±1.8 yr) with no clinical evidence of COPD (Table 1), and 20 COPD patients (15 men and 5 women; 62.9±2.7 yr; *P*>0.05) with clinical and spirometric evidence of COPD (Table 1). Patients and Control participants were studied at the Cardiothoracic Surgery department at the Second Xiangya Hospital, China. COPD patients were with a post-bronchodilator forced expiratory volume in one second to vital capacity ratio (FEV1/FVC) <0.7 (1). COPD patients were with smoking history [(33.8±3.2) pack-years], and Control participants were with little smoking history [(12.1±1.1) pack-years; *P*<0.05]. 

Serum was obtained from 15 Control participants (12 men and 3 women; 62.4±1.3 yr) with no clinical evidence of COPD and carcinoma (Table 2), and 25 COPD patients (20 men and 5 women; 63.9±1.7 yr; *P*>0.05) with clinical and spirometric evidence of COPD and with no carcinoma (Table 2). Patients and Control participants were studied at the Division of Pulmonary and Critical Care Medicine of Second Xiangya Hospital, China. COPD patients were with a smoking history [(36.2±2.2) pack-years], and the Control participants were with little smoking history [(13.1±1.3) pack-years; *P*<0.05]. 

Exclusion criteria: AECOPD or pulmonary infection within 8 weeks before this research, tuberculosis, bronchiectasis, asthma, pulmonary resection, malignancy of other systems within past 5 years, severe pain syndromes that could interfere with physical activity, antibiotics and glucocorticoids taken before the operation. 

***Lung tissues samples preparation in emphysematous mice***

Lung tissue was inflated and fixed in 10% neutral buffered formalin at a constant pressure of 25 cm H_2_O for 24 hr, and then paraffin sections were cut at 5 μm and used for pathology. Other lung samples were homogenized in lysis buffer immediately, and protein concentrations of supernatants were detected by BCA protein assay (Beyotime Institute of Biotechnology, Shanghai, CN).

***Morphological assessment in emphysematous mice***

Hematoxylin and eosin (HE) were used for staining of the sections, and morphological assessments were observed in a blinded fashion. 

Mean linear intercept (MLI) (5) was evaluated by dividing length of a line drawn across section by total number of intercepts encountered at a magnification of 100×. Destructive index (DI) (5) was measured by dividing the number of destroyed alveoli by total number of the counted alveoli at a magnification of 100×. Mean alveolar septum thickness (MAST) (5) was estimated by averaging 400 measurements per 10 representative fields at a 400× magnification.

***Apoptosis assay in emphysematous mice***

Apoptosis detection was performed by TUNEL assay (*In-situ* Apoptosis Detection Kit; Nanjing Keygen Biotech. CO., LTD, Nanjing, Jiangsu, CN). Percentage of positive nuclei out of total of 3,000 nuclei^5 ^was defined as apoptosis index (AI). 

***Immunohistochemistry for localization and expression of endothelial cell specific molecule-1 in emphysematous mice***

Tissue sections were incubated with goat polyclonal antibody corresponding to ESM-1 (1:100, R&D Systems Inc., Minneapolis, Minnesota, USA). Then, Polink-2 HRP Plus Goat Detection System (Zhongshan Goldebridge Biotechnology CO., LTD, Beijing, CN) and DAB Detection Kit (Zhongshan Goldebridge Biotechnology CO., LTD, Beijing, CN) were used for examination. Expression of ESM-1 was detected at a magnification of 200×. Integral optical density/Area (AOD) was analyzed by Image-Pro Plus7.0 (Media Cybernetics, Inc., Bethesda, Maryland, USA).

***Measurements of serum endothelial cell specific molecule-1 concentration in emphysematous mice***

ESM-1 concentrations were measured in serum of all participants using ELISA Kit (Abcam Plc., Cambridge, Cambridgeshire, UK) with a sensitivity of 1 pg/ml and no cross-reactivity against other cytokines. 

***Western blotting for expression of endothelial cell specific molecule-1, hepatocyte growth factor and vascular endothelial growth factor in emphysematous mice***

Expression of ESM-1 (20 kDa), HGF (91 kDa) and VEGF (23 kDa) were detected in lung homogenates. Homogenate supernatants were resolved and transferred onto PVDF membranes, incubated with goat polyclonal antibody against ESM-1 (1:1500, R&D Systems Inc., Minneapolis, Minnesota, USA), or rabbit polyclonal antibody against HGF (1:300, Santa Cruz Biotechnology, Inc., Santa Cruz, California, USA), or mouse monoclonal antibody against VEGF (1:300, Santa Cruz Biotechnology, Inc., Santa Cruz, California, USA) for 2 hr at 37 ^°^C. Then, membranes were washed and incubated with secondary antibody (Beyotime Institute of Biotechnology, Shanghai, CN). Proteins were evaluated by Western blotting detection reagents (Immobilon-P, Millipore, Bredford, Massachusetts, USA). Exposures were quantified with Quantity One 1-D (Bio-Rad Laboratories, Hercules, California, USA). Relative density of the protein to β-actin was calculated as expression of labeled proteins.

***Lung tissues samples preparation in human***

Lung tissues were fixed in formalin, cut into 5 μm thick sections and used for pathology. Other tissues were homogenized in lysis buffer, and protein concentrations were detected by BCA protein assay (Beyotime Institute of Biotechnology, Shanghai, CN).

***Morphological assessment in human***

Lung tissue sections were stained with HE. MLI, DI and MAST were quantified as previously described (5). 

***Apoptosis assay in human***

Apoptosis was detected by TUNEL assay (*In-situ* Apoptosis Detection Kit; Nanjing Keygen Biotech. CO., LTD, Nanjing, Jiangsu, CN), and AI was then calculated (5). 

***Immunohistochemistry for localization and expression of endothelial cell specific molecule-1 in human***

Sections were incubated with anti-ESM-1 (goat polyclonal antibody, 1:100, R&D Systems Inc., Minneapolis, Minnesota, USA) to detect ESM-1 expression. Examination method and AOD were performed as previously described.

***Measurements of serum endothelial cell specific molecule-1 concentration in human***

ESM-1 concentrations were measured in serum of all participants using ELISA Kit (Abcam Plc., Cambridge, Cambridgeshire, UK). 

***Western blotting for expression of endothelial cell specific molecule-1, hepatocyte growth factor and vascular endothelial growth factor in human***

Expression of ESM-1 (20 kDa), HGF (91 kDa) and VEGF (23 kDa) were detected in lung homogenates as previously described. The labeled proteins were normalized to β-actin.

***Statistical analysis***

SPSS 19.0 (SPSS Inc., Chicago, Illinois, USA) was used for statistical analyses of data with double blindness. Shapiro–Wilk test tested data distribution. Continuous data were expressed as mean±standard deviation (SD). Independent-Samples T test evaluated differences among two groups. Pearson correlation analysis analyzed the correlation of variables. *P *values for significance were set at <0.05.

## Results

***Morphological findings in the lungs***

**Figure 1:**
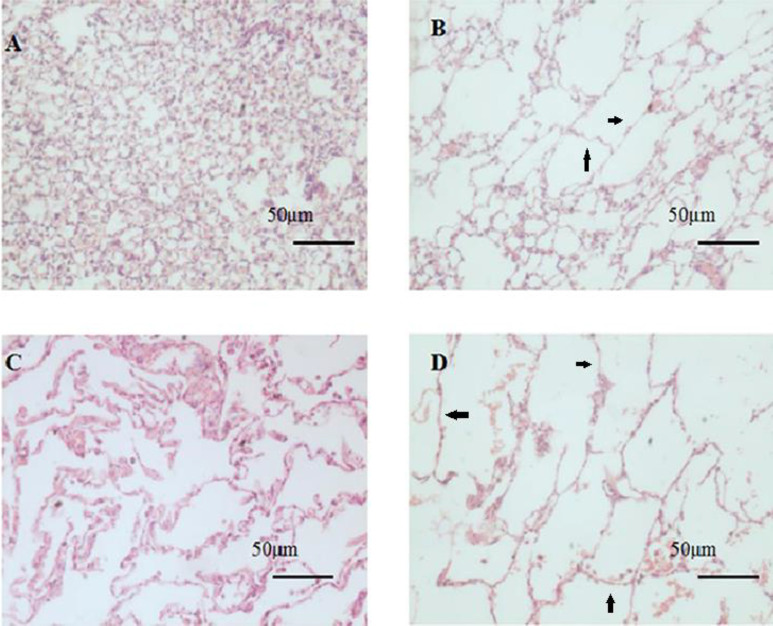
Pathology of pulmonary sections stained with hematoxylin and eosin

The lung tissues of PBS-group/Control participants were normal (Figure 1-A,C). Alveolus destruction and inflammatory infiltration were observed in lung sections of CSE-group/COPD patients (Figure 1-B,D). MLI and DI were upregulated and MAST was reduced significantly in CSE-group/COPD patients compared to values in PBS-group/Control participants (*P*<0.05; Table 3, 4). 

***Apoptosis in the lungs***

**Figure 2:**
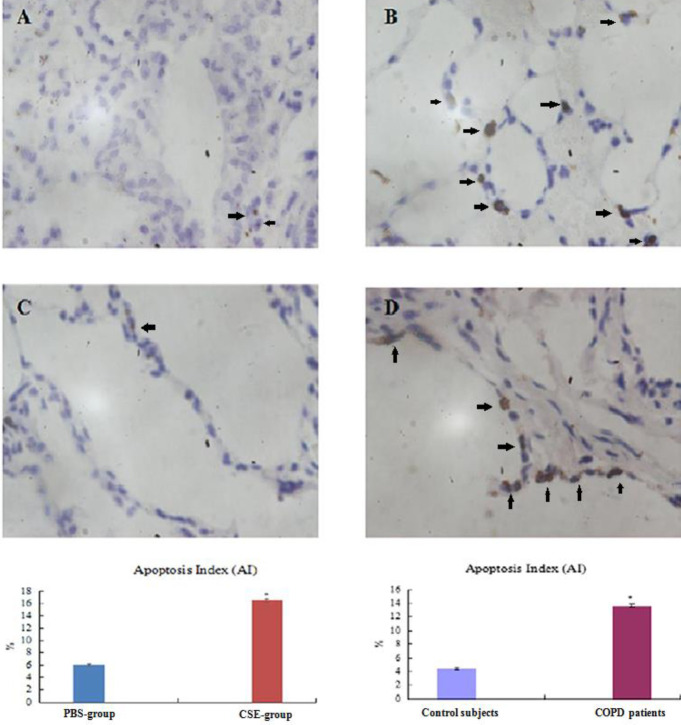
TUNEL staining detects apoptotic nuclei

As shown in Figure 2, nuclei of positive cells were brown. AI in CSE-group/COPD patients were elevated compared to PBS-group/Control participants (*P*<0.05, Figure 2; Table 5, 6). 

***Location and expression of endothelial cell specific molecule-1 in lungs***

**Figure 3:**
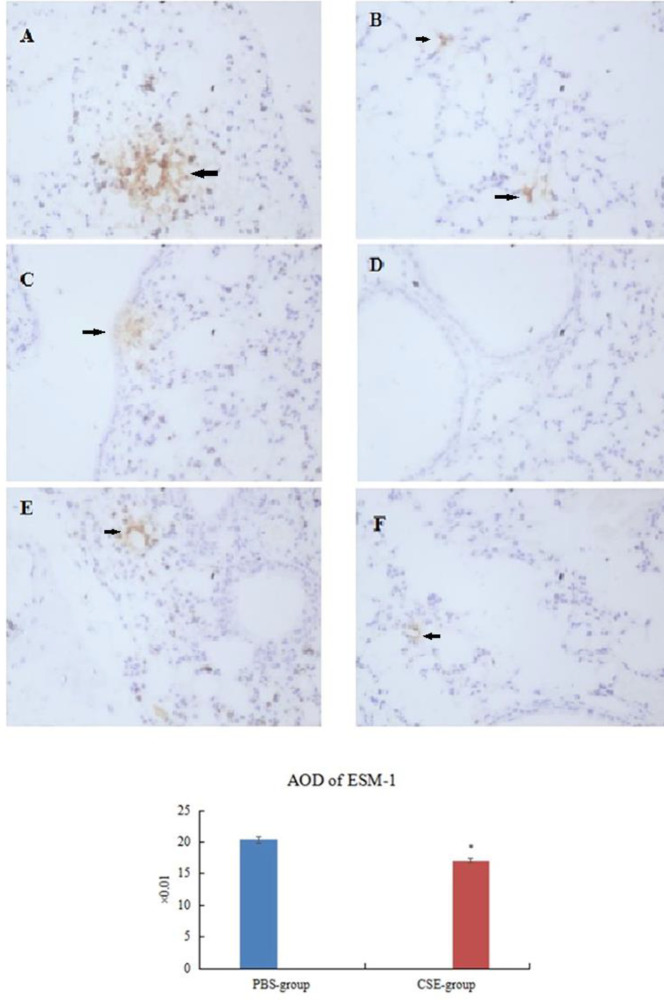
Expressin of endothelial cell specific molecule-1 in lung tissue (immunohistochemistry)

**Figure 4:**
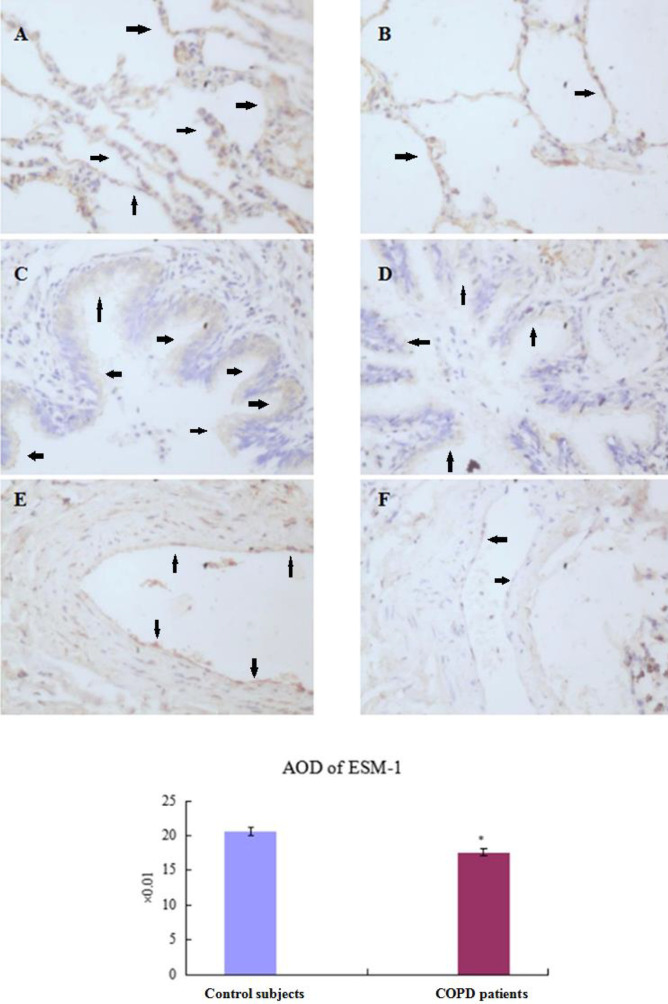
Expression of endothelial cell specific molecule-1 in lung tissue (immunohistochemistry)

The cytoplasm of positive cells was brown, and frequently localized in septal structures with alveolar epithelial cells and capillary endothelial cells in dominance in PBS-group/Control participants (Figure 3, 4). Expression of ESM-1decreased significantly in CSE-group/COPD patients (*P<*0.05, Figure 3, 4; Table 5, 6). 

***Endothelial cell specific molecule-1 level in serum***

**Figure 5:**
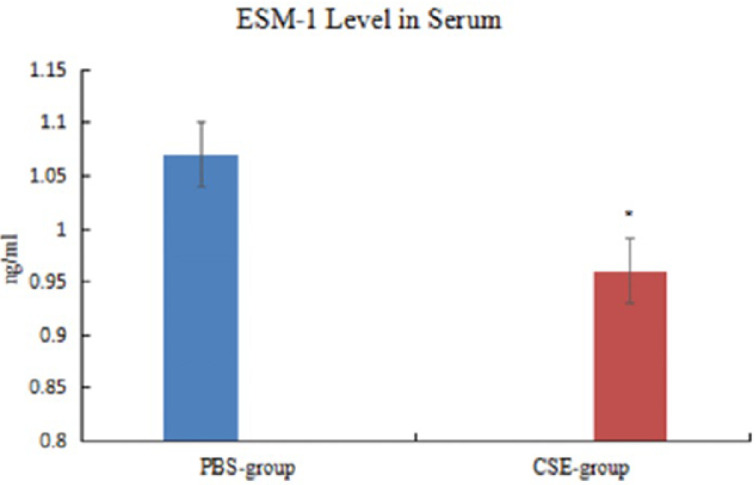
Level of endothelial cell specific molecule-1 in serum of mice

**Figure 6:**
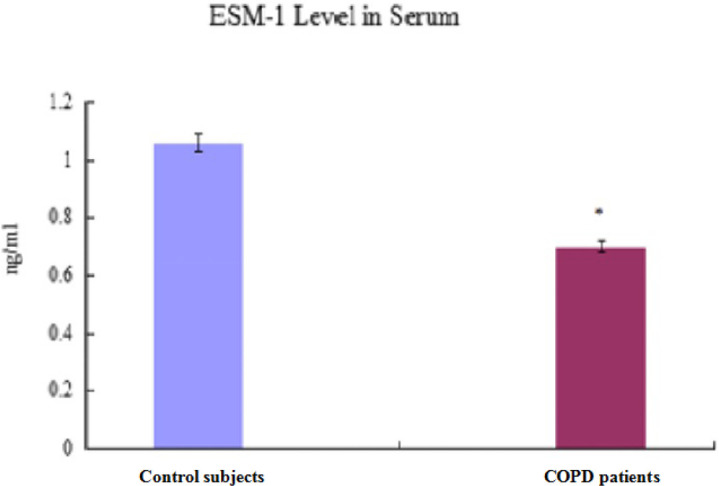
Endothelial cell specific molecule-1 level in serum of human

Compared with PBS-group/Control participants, ESM-1 level in serum decreased significantly in CSE-group/COPD patients (*P<*0.05, Figure 5, 6; Table 5, 7). 

***Western blotting assay of endothelial cell specific molecule-1, hepatocyte growth factor, and vascular endothelial growth factor in the lungs***

**Figure 7:**
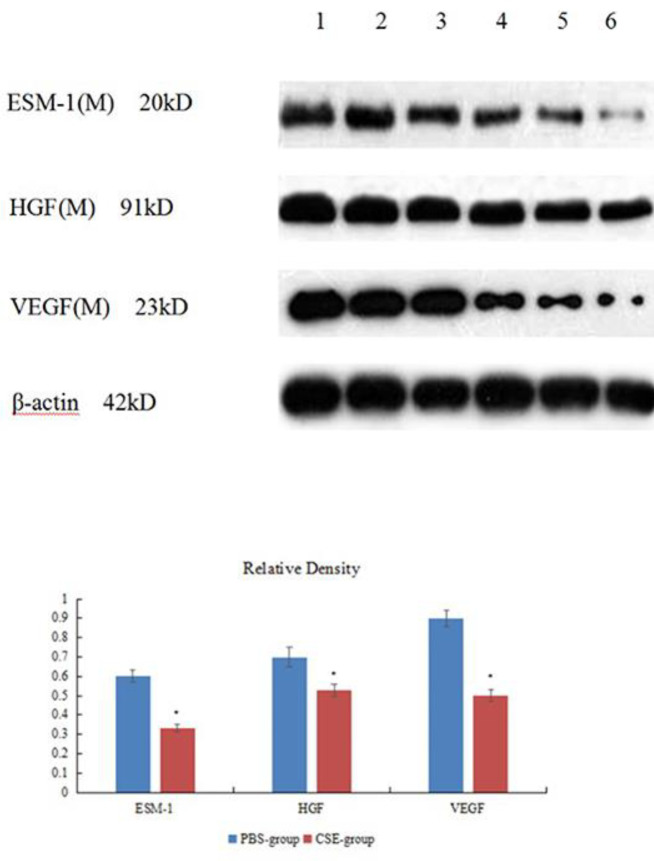
Expression of endothelial cell specific molecule-1, hepatocyte growth factor, and vascular endothelial growth factor (Western blotting) in lung tissue of mice

**Figure 8:**
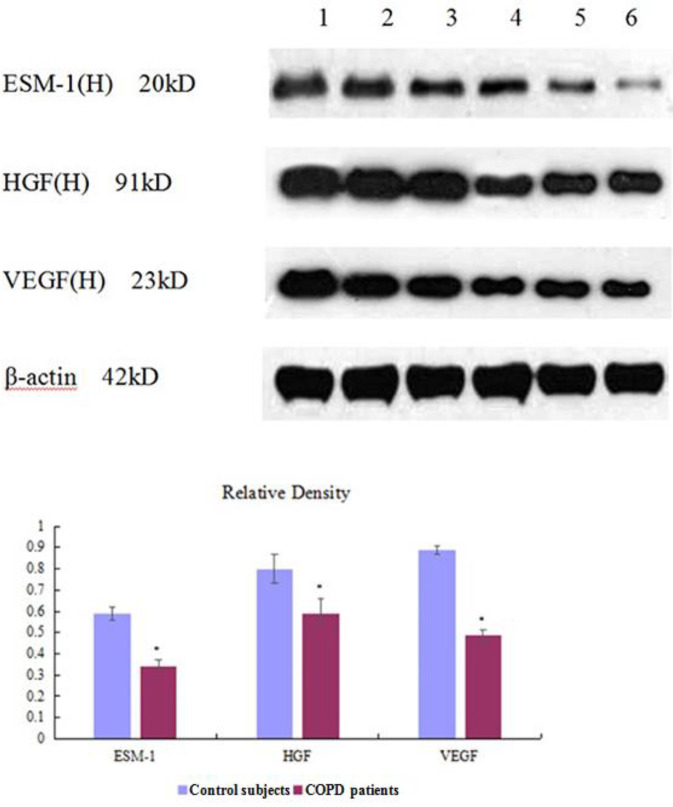
Expression of endothelial cell specific molecule-1, hepatocyte growth factor, and vascular endothelial growth factor (Western blotting) in lung tissue of human

Protein expressions in the lungs from all experimental groups were measured by Western blotting (Figure 7, 8). Expression of ESM-1, HGF, and VEGF in CSE-group/COPD patients were lower than in PBS-group/Control participants (*P<*0.01, Table 8, 9). 

***Correlation analysis***

Correlations between AI and concentration of ESM-1 in serum, as well as expression of ESM-1 in lung tissue, which was detected by western blotting, in mice were all negative (r=-0.774, *P*<0.05; *r*=-0.743, *P*<0.001). Correlation between ESM-1 and VEGF, and HGF in lung tissue in mice were all positive (*r*=0.898, *P*<0.01; *r*=0.768, *P*<0.01) . 

Correlations between AI of pulmonary structural cells and concentration of ESM-1 in serum, and the expression of ESM-1 in lung tissue, which was detected by western blotting, in human were all negative (*r*=-0.744, *P*<0.01; *r*=-0.798, *P*<0.01). Correlation between ESM-1 and VEGF, and HGF in lung tissue in human were all positive (*r*=0.795, *P*<0.01; *r*=0.787, *P*<0.01).

**Table 1:** Pulmonary function examined from participants undergoing lung resection (mean±standard deviation)

Forced respiratory volume in the first second% (FEV1%）	
Control participants（n=12）	（110.80±8.25）%
COPD patients （n=20）	（66.20±8.56）%

**Table 2:** Pulmonary function examined from participants undergoing serum draw (mean±standard deviation)

Forced respiratory volume in the first second% (FEV1%）	
Control subjects（n=15）	（102.31±7.85）%
COPD patients （n=25）	（63.20±6.38）%

The Figure demonstrated alveolar structure in A: PBS-group (n=7), B: CSE-group (n=14), C: Control participants (n=12), D: COPD patients (n=20). 100x magnification. COPD: Chronic obstructive pulmonary disease; CSE: Cigarette smoke extract; PBS: phosphate-buffered saline

**Table 3:** Morphological assessment in lung tissue sections in mice(mean±standard deviation)

Group	MLI (μm)	DI (%)	MAST (μm)
PBS-group (n=7)	21.4±0.9	12.6±1.3	9.6±0.5
CSE-group (n=14)	40.1±1.4*	36.0±1.5*	4.3±0.3*

*P<0.05 in comparison with PBS-group. CSE: Cigarette smoke extract; MLI: Mean linear intercept; DI: Destructive index; MAST: Mean alveolar septum thickness; PBS: phosphate-buffered saline

**Table 4:** Morphological assessment in lung tissue sections in human(mean±standard deviation)

Group	MLI (μm)	DI (%)	MAST (μm)
Control subjects (n=12)	34.1±2.9	16.1±1.2	32.0±1.7
COPD patients (n=20)	54.0±6.0^*^	43.4±2.1^*^	18.5±2.1^*^

*P<0.05 in comparison with Control participants. COPD: Chronic obstructive pulmonary disease; MLI: Mean linear intercept; DI: Destructive index; MAST: Mean alveolar septum thickness

The Figure showed apoptotic cells in the A: PBS-group (n=7), B: CSE-group (n=14), C: Control participants (n=12), D: COPD patients (n=20). 400x magnification. Values are means±SD. Significant differences were demonstrated by Independent-Samples T test. Measured value of AI (%): *P<0.05 in comparison with PBS-group/Control participants. COPD: Chronic obstructive pulmonary disease; CSE: Cigarette smoke extract; AI: Apoptosis index; PBS: phosphate-buffered saline

**Table 5:** Apoptosis and expression of endothelial cell specific molecule-1 in lung tissues and serum in mice (mean±standard deviation)

Group	AI (%)	AOD of ESM-1	ESM-1 in serum (ng/ml)
PBS-group (n=7)	5.99±0.16	0.204±0.005	1.07±0.03
CSE-group (n=14)	16.50±0.25^*^	0.171±0.003^*^	0.96±0.03^*^

*P<0.05 in comparison with PBS-group. ESM-1: Endothelial cell specific molecule-1; CSE: Cigarette smoke extract; AI: Apoptosis index; PBS: phosphate-buffered saline; AOD: Integral optical density/Area

The Figure showed expression of ESM-1 in the A, C, E: PBS-group (n=7), and B, D, F: CSE-group (n=14). 200x magnification. Values are means±SD. Significant differences were determined by Independent-Samples T test. Measured value of AOD: *P<0.05 in comparison with PBS-group. ESM-1: Endothelial cell specific molecule-1; CSE: Cigarette smoke extract; PBS: phosphate-buffered saline; AOD: Integral optical density/Area

**Table 6:** Apoptosis and expression of endothelial cell specific molecule-1 in lung tissues of human (mean±standard deviation)

Group	AI (%)	AOD of ESM-1
Control subjects (n=12)	4.33±0.15	0.206±0.006
COPD patients (n=20)	13.62±0.22^*^	0.176±0.005^*^

*P<0.05 in comparison with Control participants. COPD: Chronic obstructive pulmonary disease; ESM-1: Endothelial cell specific molecule-1; AI: Apoptosis index; AOD: Integral optical density/Area

Findings showed expression of ESM-1 in the A, C, E: Control participants (n=12), and B, D, F: COPD patients (n=20). 200x magnification. Values are means±SD. Significant differences were determined by Independent-Samples T test. Measured value of AOD: *P<0.05 in comparison with Control participants. COPD: Chronic obstructive pulmonary disease; ESM-1: Endothelial cell specific molecule-1; AOD: Integral optical density/Area

PBS-group (n=7), CSE-group (n=14). Values are means±SD. Significant differences were determined by Independent-Samples T test. Concentration: *P<0.05 in comparison with PBS-group. ESM-1: Endothelial cell specific molecule-1; CSE: Cigarette smoke extract; PBS: phosphate buffer saline

Control participants (n=15), COPD patients (n=25). Values are means±SD. Significant differences were calculated by Independent-Samples T test. Concentration: *P<0.05 in comparison with Control participants. COPD: Chronic obstructive pulmonary disease; ESM-1: Endothelial cell specific molecule-1

**Table 7:** Expression of endothelial cell specific molecule-1 in serum of human (mean±standard deviation)

Group	ESM-1 in serum (ng/ml)
Control participants (n=15)	1.06±0.03
COPD patients (n=25)	0.70±0.02^*^

*P<0.05 in comparison with Control participants. COPD: Chronic obstructive pulmonary disease; ESM-1: Endothelial cell specific molecule-1

**Table 8:** Relative density of endothelial cell specific molecule-1, hepatocyte growth factor, and vascular endothelial growth factor in the lungs in mice (mean±standard deviation)

Group	ESM-1	HGF	VEGF
PBS-group (n=7)	0.60±0.03	0.70±0.05	0.90±0.04
CSE-group (n=14	0.33±0.02^*^	0.53±0.03^*^	0.50±0.03^*^

*P<0.01 in comparison with PBS-group. VEGF: Vascular endothelial growth factor; HGF: Hepatocyte growth factor; ESM-1: Endothelial cell specific molecule-1; CSE: Cigarette smoke extract; PBS: phosphate-buffered saline

PBS-group (n=7), CSE-group (n=14). Lane 1, 2, 3 were from PBS-group, Lane 4, 5, 6 were from CSE-group. Values are means±SD. Significant differences were determined by Independent-Samples T test. Measured value of Ratio: *P<0.01 in comparison with PBS-group. VEGF: Vascular endothelial growth factor; HGF: Hepatocyte growth factor; ESM-1: Endothelial cell specific molecule-1; CSE: Cigarette smoke extract; PBS: phosphate-buffered saline

**Table 9:** Relative density of endothelial cell specific molecule-1, hepatocyte growth factor, and vascular endothelial growth factor in the lungs in human (mean±standard deviation)

Group	ESM-1	HGF	VEGF
Control participants (n=12)	0.59±0.03	0.80±0.07	0.89±0.02
COPD patients (n=20)	0.34±0.02^*^	0.59±0.05^*^	0.49±0.04^*^

*P<0.01 in comparison with control participants. COPD: Chronic obstructive pulmonary disease; VEGF: Vascular endothelial growth factor; HGF: Hepatocyte growth factor; ESM-1: Endothelial cell specific molecule-1

Control participants (n=12), COPD patients (n=20). Lane 1, 2, 3 were from control participants, Lane 4, 5, 6 were from COPD patients. Values are means±SD. Significant differences were determined by Independent-Samples T test. Measured value of Ratio: *P<0.01 in comparison with control participants. COPD: Chronic obstructive pulmonary disease; VEGF: Vascular endothelial growth factor; HGF: Hepatocyte growth factor; ESM-1: Endothelial cell specific molecule-1

## Discussion

To our knowledge, the current study is the first to show markedly decreased ESM-1 level in emphysematous mice and stable COPD patients. Our results are contrary to the two articles (13, 14) that have been reported. Also, our findings suggested reducing expression of VEGF and HGF in lungs of emphysematous mice and stable COPD patients. The present study suggested a close relationship between decreased ESM-1 levels and elevated cell apoptosis in emphysematous lungs. 

Chronic inflammation of airways and progressive destruction of pulmonary parenchyma are characteristics of COPD. The pathophysiological mechanisms of COPD are complex and have not yet been fully clarified. Apoptosis of pulmonary structural cells is one of the important pathogenesis of COPD (1-5). ESM-1 has been implicated in adhesion, migration and proliferation (10, 11). ESM-1 has been observed in the cytoplasm of endothelium and epithelium of lung (8). Previous reports mentioned that ESM-1 plays a role in endothelial proliferation (15-19). Since COPD is a disease with abnormal pulmonary structural cell apoptosis (1-5), decrease of ESM-1 is expected. So, decreased ESM-1 level was shown in our study.

This study demonstrates the different role of ESM-1 in COPD. Our results are contrary to the two existing articles (13,14). Pihtili, *et al. *(13) showed that serum ESM-1 level was higher in stable COPD patients. Dai, *et al*. (14) reported that plasma ESM-1 levels in COPD patients were higher than in healthy people; hence, inhibition of ESM-1 may reduce apoptosis in COPD. Our study showed for the first time that expression of the ESM-1 decreased in emphysematous mice and stable COPD patients. In this study, apoptotic alveolar epithelium and endothelium increased in the lungs of emphysematous mice and stable COPD patients. Correlation between AI and ESM-1 was negative. So, we surmised that reduction of ESM-1 expression would induce destruction and insufficient repair of lung tissue, leading to development of emphysema. ESM-1 seems to be a potential molecule for anti-apoptotic therapy. 

ESM-1 is related with many diseases. It effectively relieved pulmonary epithelium apoptosis caused by LPS exposure (15). Low ESM-1 levels are predictive of ARDs in severe sepsis and septic shock (16). Downregulation of ESM-1 in myeloid leukemia cells inhibits proliferation and promotes apoptosis (17). ESM-1 silencing induces programmed cell death in hepatocarcinoma (18). ESM-1 has been reported to be over-expressed in lung cancer and significantly upregulated in malignant pleural effusion (19). Thus, these studies suggested that inhibition of ESM-1 was not a suitable therapeutic strategy for apoptosis, and this was consistent with our findings.

ESM-1 is secreted by vascular endothelium and epithelium lining distal bronchi, and lung submucosal glands (8). ESM-1 interacts with HGF through its glycan chain (9). HGF is also a crucial cell factor for the lung repair in COPD, which reverses alveolar regeneration and restoration of pulmonary function affected by emphysema (7, 20, 21). ESM-1 dose-dependently increases HGF-mediated proliferation of cells (9). In this study, expression of HGF decreased significantly in emphysematous mice and stable COPD patients. Decreased expression of ESM-1 led to a concomitant increased apoptosis of structural cells in emphysematous lungs. ESM-1 levels declined in serum of emphysema mice and stable COPD patients. There was positive correlation between expression of HGF and ESM-1. So, we surmised that expression of ESM-1 protein would influence HGF-mediated repair of lung tissue in COPD. ESM-1 is one of the genes that were most potently induced by VEGF (12). VEGF plays an important part in alveolar structural maintenance and lung development (22). Airway administration of VEGF siRNAs induces transient air space enlargement in the emphysema model of mice (23). VEGF decreases in smoking-induced COPD model in rats (24). VEGF levels are significantly lower in smokers with COPD than in non-smokers (25). Low VEGF level plays an important role in trigger of pulmonary structural cells apoptosis in COPD (26, 27). Our research showed that VEGF reduced in emphysema mice and stable COPD patients, and there is negative correlation between the contents of VEGF and AI of the alveolar septal cell. There was also positive correlation between expression of VEGF and ESM-1. The expression of ESM-1 may influence anti-apoptotic role of VEGF in COPD. As ESM-1 interacts with HGF and VEGF, we confirmed that low ESM-1 levels induce the apoptosis of pulmonary structural cells in stable COPD.

Although ESM-1 plays some role in the process of stable COPD, the relationship may be more complex, including factors such as differential COPD GOLD categories, differences in lung function tests, and differences in sample and response to available therapeutic modalities. 

There were some limitations in our study. One of limitations was the small sample size of patients, wherein only patients in stable period were included, and the changes of ESM-1 in acute exacerbation stage were not observed. Emphysematous mice tests had been carried out, but therapeutic interventions of ESM-1 were still needed to determine its effect on COPD cell apoptosis more clearly *in vivo *and* in vitro*.

## Conclusion

We showed that expression of ESM-1 decreased in emphysematous mice and stable COPD patients, at protein levels, and the results suggest that ESM-1 may play anti-apoptotic role in the process of COPD. We believe that further studies should aim to research on ESM-1, which may lead to new therapeutic targets in COPD.

## Data Availability

The datasets analyzed in this research are available from corresponding author on reasonable request.

## References

[1] Ozretić P, da Silva Filho MI, Catalano C, Sokolović I, Vukić-Dugac A, Šutić M (2019). Association of NLRP1 coding polymorphism with lung function and serum IL-1β concentration in patients diagnosed with chronic obstructive pulmonary disease (COPD). Genes (Basel).

[2] Chen L, Luo L, Kang N, He X, Li T, Chen Y (2020). The protective effect of HBO1 on cigarette smoke extract-induced apoptosis in airway epithelial cells. Int J Chron Obstruct Pulmon Dis.

[3] He X, Li T, Kang N, Zeng H, Ren S, Zong D (2017). The protective effect of PRMT6 overexpression on cigarette smoke extract-induced murine emphysema model. Int J Chron Obstruct Pulmon Dis.

[4] Shi Z, Chen Y, Cao J, Zeng H, Yang Y, Chen P (2017). Intratracheal transplantation of endothelial progenitor cells attenuates smoking-induced COPD in mice. Int J Chron Obstruct Pulmon Dis.

[5] Zhang Y, Cao J, Chen Y, Chen P, Peng H, Cai S (2013). Intraperitoneal injection of cigarette smoke extract induced emphysema, and injury of cardiac and skeletal muscles in BALB/C mice. Exp Lung Res.

[6] Lee KH, Lee CH, Jeong J, Jang AH, Yoo CG (2015). Neutrophil Elastase Differentially Regulates Interleukin 8 (IL-8) and Vascular Endothelial Growth Factor (VEGF) Production by Cigarette Smoke Extract. J Biol Chem.

[7] Calvi C, Podowski M, Lopez-Mercado A, Metzger S, Misono K, Malinina A (2013). Hepatocyte growth factor, a determinant of airspace homeostasis in the murine lung. PLoS Genet.

[8] Tsai JC, Zhang J, Minami T, Voland C, Zhao S, Yi X (2002). Cloning and characterization of the human lung endothelial-cell-specific molecule-1 promoter. J Vasc Res.

[9] Béchard D, Gentina T, Delehedde M, Scherpereel A, Lyon M, Aumercier M (2001). Endocan is a novel chondroitin sulfate/dermatan sulfate proteoglycan that promotes hepatocyte growth factor/scatterfactormitogenic activity. J Biol Chem.

[10] Afsar B, Takir M, Kostek O, Covic A, Kanbay M (2014). Endocan: a new molecule playing a role in the development of hypertension and chronic kidney disease?. J Clin Hypertens (Greenwich).

[11] Shin JW, Huggenberger R, Detmar M (2008). Transcriptional profiling of VEGF-A and VEGF-C target genes in lymphatic endothelium reveals endothelial-specific molecule-1 as a novel mediator of lymphangiogenesis. Blood.

[12] Li C, Geng H, Ji L, Ma X, Yin Q, Xiong H (2019). ESM-1: A Novel tumor biomaker and its research advances. Anticancer Agents Med Chem.

[13] Pihtili A, Bingol Z, Kiyan E (2018). Serum endocan levels in patients with stable COPD. Int J Chron Obstruct Pulmon Dis.

[14] Dai L, He J, Chen J, Wang T, Liu L, Shen Y (2018). The association of elevated circulating endocan levels with lung function decline in COPD patients. Int J Chron Obstruct Pulmon Dis.

[15] Zhang X, Zhuang R, Wu H, Chen J, Wang F, Li G (2018). A novel role of endocan in alleviating LPS-induced acute lung injury. Life Sci.

[16] Gaudet A, Parmentier E, Dubucquoi S, Poissy J, Duburcq T, Lassalle P (2018). Low endocan levels are predictive of Acute Respiratory Distress Syndrome in severe sepsis and septic shock. J Crit Care.

[17] Sun L, Sun C, Sun J, Yang W (2019). Downregulation of ENDOCAN in myeloid leukemia cells inhibits proliferation and promotes apoptosis by suppressing nuclear factorκB activity. Mol Med Rep.

[18] Yang J, Sheng S, Yang Q, Li L, Qin S, Yu S (2017). Endocan silencing induces programmed cell death in hepatocarcinoma. Oncol Lett.

[19] Lu GJ, Shao CJ, Zhang Y, Wei YY, Xie WP, Kong H (2017). Diagnostic and prognostic values of endothelial-cell-specific molecule-1 with malignant pleural effusions in patients with non-small cell lung cancer. Oncotarget.

[20] Kennelly H, Mahon BP, English K (2016). Human mesenchymal stromal cells exert HGF dependent cytoprotective effects in a human relevant pre-clinical model of COPD. Sci Rep.

[21] Cho RJ, Kim YS, Kim JY, Oh YM (2017). Human adipose-derived mesenchymal stem cell spheroids improve recovery in a mouse model of elastase-induced emphysema. BMB Rep.

[22] Jiménez J, Lesage F, Richter J, Nagatomo T, Salaets T, Zia S (2018). Upregulation of vascular endothelial growth factor in amniotic fluid stem cells enhances their potential to attenuate lung injury in a preterm rabbit model of bronchopulmonary dysplasia. Neonatology.

[23] Takahashi Y, Izumi Y, Kohno M, Ikeda E, Nomori H (2013). Airway administration of vascular endothelial growth factor siRNAs induces transient airspace enlargement in mice. Int J Med Sci.

[24] Wan YF, Huang ZH, Jing K, Li J, Wang Y, Xu CQ (2015). Azithromycin attenuates pulmonary inflammation and emphysema in smoking-induced COPD model in rats. Respir Care.

[25] Kawamoto T, Kanazawa H, Tochino Y, Kawaguchi T (2018). Evaluation of the severity of small airways obstruction and alveolar destruction in chronic obstructive pulmonary disease. Respir Med.

[26] Prakash Muyal J, Kumar D, Kotnala S, Muyal V, Kumar Tyagi A (2015). Recombinant human keratinocyte growth factor induces Akt mediated cell survival progression in emphysematous mice. Arch Bronconeumol.

[27] Sun Y, An N, Li J, Xia J, Tian Y, Zhao P (2019). miRNA-206 regulates human pulmonary microvascular endothelial cell apoptosis via targeting in chronic obstructive pulmonary disease. J Cell Biochem.

